# Palliative care needs of patients with multiple sclerosis in southeast Iran

**DOI:** 10.1186/s12904-021-00867-3

**Published:** 2021-10-28

**Authors:** Fatemeh Dadsetan, Parvin Mangolian Shahrbabaki, Moghadameh Mirzai, Esmat Nouhi

**Affiliations:** 1 M.s Medical Surgical Nursing, Nursing Research Center, Kerman University of Medical Sciences, Kerman, Iran; 2 Nursing Research Center, Kerman University of Medical Sciences, Kerman, Iran; 3 Razi Faculty of Nursing and Midwifery, Department of Critical Care Nursing, Kerman University of Medical Sciences, Kerman, Iran; 4 Health Modeling Research Center, Kerman University of Medical Sciences, Kerman, Iran; 5 Razi Faculty of Nursing and Midwifery, Department of Medical Surgical Nursing, Kerman University of Medical Sciences, Kerman, Iran

**Keywords:** Nurse, Patient, Viewpoint, Palliative need, Multiple sclerosis

## Abstract

**Background:**

Due to the chronic nature of multiple sclerosis, palliative care can play a significant role in improving the quality of life and well-being of the affected patients. An essential step for developing appropriate palliative care for these patients is to determine the types of palliative care necessary, from different points of view. Therefore, this study was conducted to compare the palliative care needs from the nurses’ and patients’ points of view in southeast Iran in 2017.

**Method:**

This descriptive-analytical cross-sectional study was conducted on 154 nurses working in neurology wards of teaching hospitals associated with Kerman University of Medical Sciences and 132 patients with multiple sclerosis who were referred to these hospitals in southeast Iran. The data were collected using a questionnaire for assessing the palliative care needs of patients with multiple sclerosis. Pearson correlation coefficient, independent *t*-test, ANOVA, chi-square, and the Mann-Whitney and Kruskal-Wallis tests were used to examine the data.

**Results:**

Both nurses and patients mentioned the palliative needs of patients with multiple sclerosis in terms of physical, social, spiritual, psychological, and economic dimensions, respectively, but the results showed that there was a significant difference between the two groups in all dimensions of palliative needs (*P* < 0.0001).

**Conclusion:**

Given the differences in how patients and nurses prioritize palliative care needs, it is essential to consider the different dimensions of palliative needs of patients with multiple sclerosis.

## Background

Multiple sclerosis is a progressive chronic disease of the central nervous system. In this disease, the myelin layer that insulates nerve fibers is damaged, and messages from the nervous system become slow or blocked [1]. Research shows that 3.5–5% of people with multiple sclerosis are below 18 [2]. Multiple sclerosis leads to death in more than 50% of patients affected by it. The incidence of deaths caused by multiple sclerosis varies from country to country [3]. Standardized prevalence and incidence in Europe are 101.8 and 4.44 per 100,000 people, respectively. In Iran, the prevalence is 54.51 and the incidence is 5.87 per 100,000 people. The prevalence of multiple sclerosis in southeastern Iran is in the medium range [4, 5].

The proportion of non-cancer patients currently being referred to palliative care has increased; these patients are younger, and although they are less likely to be admitted to the hospital, they have complicated problems [6]. Despite improvements in palliative care, access to palliative care remains inconsistent, even in high-income countries, with the number of providers varying from 5 to 680 per million of the population. Overall, only a small proportion of those who need palliative care, perhaps as low as 14%, receive it [7].

Palliative care is an approach that addresses the quality of patient and family life by identifying needs and treating physical, spiritual, mental, and social problems [8]. The needs related to palliative care are diverse concerning age, cultural background, supportive systems, and degree of disease progression [9]. In palliative care, nurses try to create a structured way of providing care and giving patients a good feeling [10]. While living with multiple sclerosis is a challenge, palliative care can make a big difference. Some palliative care treatments for MS include medications and techniques to relieve muscle spasms, calm nerves, ease depression, and manage pain [11]. Adjusting to an MS diagnosis can also be very difficult, not only for patients but also for their families. However, in addition to treating the symptoms of multiple sclerosis, palliative care specialists can also support patients and their families in many ways; they can help patients adjust to their diagnosis and help them match their treatment choices to their personal needs and goals [12].

Patients with multiple sclerosis are typically not familiar with palliative care, and even if they are, they rarely consider it for themselves as they think it is only meant for patients with cancer [11, 13] These patients avoid palliative care possibly because palliative care for MS patients appears counterintuitive to many providers even though some countries, such as the UK, have played a pioneering role in implementing palliative care for patients with long-term neurological conditions [13]. Palliative care is provided by a team of palliative care doctors, nurses, and social workers who work together with other doctors to provide an extra line of support [14]. Because nurses have a close relationship with patients, it is suggested that nurses’ knowledge, skills, and expertise support those affected by long-term conditions, including MS [15–17]. A review of the literature highlights the nurse’s critical role and provides increasing evidence that nurses can play a decisive role in providing high-quality and cost-effective health care [18].

In their study, Weissman et al. found that by implementing a screening approach for patients with the need for palliative care, they can identify the majority of these needs and provide tailored care services for complicated problems [19]. Methley et al. demonstrated in a study that needs assessment for people with multiple sclerosis requires examining the perspectives of patients and professionals [20]. Practical cooperation between professionals is essential for maintaining coordinated care and providing better care for non-cancer patients in the community [21].

It is essential to identify the palliative needs of the patient. Thus, acknowledging nurses as an influential force in the treatment process of patients and enquiring their viewpoints can further clarify the patients’ needs and provide them with a higher level of nursing care. This study aimed to compare the palliative needs of patients with multiple sclerosis from the nurses’ and patients’ points of view.

## Materials and methods

### Design and setting

This is a descriptive cross-sectional study. The study setting included neurology wards of Shahid Bahonar Hospital, affiliated with Kerman University of Medical Sciences in the southeast of Iran; this is the largest medical centers in southeastern Iran.

### Participants

The participants in this study were comprised of a group of patients and a group of healthcare professionals (nurses). The sample size calculated using the sample size formula (power = 80%, *P* = 0.05) was 132 participants recruited by the convenience sampling method [22]. The criteria for entering the study were having a definitive diagnosis of multiple sclerosis, having full awareness during the study, and being hospitalized in the neurology wards. MS can be diagnosed clinically by demonstrating two separate attacks involving at least two different areas of the central nervous system (CNS), so in order to make a diagnosis of MS, identification of evidence of damage in at least two separate areas of CNS, which includes the brain, the spinal cord, and the optic nerves, was necessary. The most recent McDonald criteria simplify the diagnostic requirements as dissemination in space (DIS) and dissemination in time (DIT). They may allow for an earlier MS diagnosis from a single baseline brain MRI if there is silent gadolinium-enhancement and non-enhancing lesions [23].

The study population consisted of 170 nurses working in the neurology wards. Inclusion criteria were having a bachelor’s degree or higher university education and at least 6 months of work experience in care for patients with multiple sclerosis in neurology wards. One hundred and fifty-four nurses participated in the study, so the response rate was 92.69%. The data of each nurse is for at least three to five patients.

### Instrument

The first part of the questionnaire was the demographic characteristics of nurses (age, gender, marital status, education, work experience, position, and training on palliative care) and patients (age, sex, marital status, education, history disease in their families, duration of the disease, and job).

Due to language barriers and cultural and religious differences, the questionnaire of palliative care needs was developed by researchers based on the literature review [24–27]. The questionnaire consisted of 48 items, and the five dimensions included physical (16 items), spiritual (8 items), psychological (8 items), social (11 items), and economic (5 items) needs. A five-point Likert scale was used (strongly disagree = 1, disagree = 2, have no opinion = 3, agree = 4, and strongly agree = 5). The range of scores was 48–240. A higher overall score meant a greater need for palliative care. The questionnaire was presented to faculty members of the nursing school with specializations in critical care, palliative care, and medical and surgical care (*n* = 10) to determine the content validity, and the content validity index (CVI) was determined as 0.91 [28]. In the current study, Cronbach’s alpha coefficient was 0.86 for the total scale, 0.89 for the physical subscale, 0.9 for the spiritual sub-scale, 0.91 for the psychological sub-scale, 0.92 for the social sub-scale, and 0.86 for the economic sub-scale.

### Data collection

Because shift nurses are in circulation, all patients are cared for by several nurses. The inclusion criteria were being over 18, fluency in the Persian language, a stay of 3 days or longer in the ward, an emergency medical status, and provision of signed consent. The sampling lasted from July 27 to October 5, and patients who did not provide written consent to enter the study or were physically and mentally unable to complete the questionnaire were excluded. The data collection was done by the first researcher.

After obtaining the permission of the Ethics Committee as well as the permits and a reference letter from the Faculty of Nursing and Midwifery and the hospitals, and the neurology wards, all eligible patients were entered into the study until the desired sample size was achieved. The first researcher identified eligible samples using the convenience sampling method and provided sufficient information about the research objectives and their importance. Aiming to better understanding, and therefor in the answers, palliative care was explained in the informed consent procedure of both the respondents’ groups.

Participants completed the questionnaire in the best physical and mental conditions possible. The completed questionnaires were received on the same shift. Data were collected at times other than during physicians’ rounds and visiting hours to ensure that data collection did not interfere with patients’ treatment and ensure that caregivers had enough time to answer the questionnaire. Data were collected when the patient was in stable condition. All participants provided informed consent, and they were assured that their personal information would remain confidential.

For nurses, a box was placed in the neurology wards so that they had enough time to complete the questionnaire and put it in the box. Because shift nurses are in circulation, the researcher went to the research setting during the morning, evening, and night shifts and provided the participants with the questionnaire if they met the inclusion criteria and were physically and psychologically fit to answer the questions. As an incentive for filling in the questionnaire, patients were trained in self-care at the request of participants, and a palliative care training workshop was held for nurses to thank them for their participation.

### Data analysis

The software used in this study was SPSS version 19. Descriptive statistics (frequency, percentage, mean, and standard deviation) were used to describe the participants’ characteristics. Skewness and kurtosis indices were used to check the normality of the data, and the independent t-test was used to compare the palliative needs of MS patients from patients’ and nurses’ points of view. The significance level was considered 0.05.

The mean age for the patients was 36.38 ± 9.38. The majority of patients were female (79.5%) and married (84.8%), were housewives (64.4%), had high school diplomas (65.9%) and no history of multiple sclerosis in their families (90.2%), and the duration of their disease was 5–10 years (35.1%) (Table 1). The mean age of the nurses was 36.38 ± 9.38. The majority of nurses were female (70.1%) and married (79.2%), had 5–10 years of experience (33.1%), and had not been trained in palliative care (84.4%) (Table 2).

**Table 1:** Demographic characteristics of multiple sclerosis patients

Variables	n	%
Age		
20–35	66	50
> 35	66	50
Gender		
Female	105	79.5
Male	27	20.5
Marital status		
Single	20	15.2
Married	112	84.8
Education		
High school or less	40	30.3
Diploma	87	65.9
B.S or more	5	3.8
History disease in their families		
No	119	90.2
Yes	13	9.8
Duration of the disease		
Less than 1 year	17	13
1–5	37	28.2
5–10	46	35.1
More than 10 years	31	23.7
Job		
Housewife	85	64.4
Employed	47	35.6

**Table 2:** Demographic characteristics of nurses

Variables	n	%
Age		
20–35	110	28.6
> 35	44	71.4
Gender		
Female	108	70.1
Male	46	29.9
Marital status		
Single	32	20.8
Married	122	79.2
Education		
M.S. and more	21	13.64
B.S	133	86.36
Work experience		
6 months to 1 year	13	8.4
1–5 years	46	29.9
5–10 years	51	33.1
More than 10 years	44	28.6
Position		
Head nurse	13	8.4
Nurse	141	91.6
Training on palliative care		
Yes	24	15.6
No	130	84.4

Palliative needs include several dimensions. The t-test showed that the palliative needs scores in the physical dimension were 42.58 ± 9.58 according to patients and 45.01 ± 8.22 according to nurses; this difference was statistically significant (*P* = 0.02). The average scores of palliative needs in the social dimension were 39 ± 6.35 from the patients’ point of view and 35.26 ± 5.1 from the nurses’ point of view, which demonstrated a statistically significant difference (*P* < 0.0001). The mean scores of palliative needs in the spiritual dimension were 31.24 ± 4.07 and 26.57 ± 3.72 from the patients’ and the nurses’ points of view, respectively, and were significantly different (*P* < 0.0001). The average scores of palliative needs in the psychological dimension were 22.31 ± 6.81 from the patients’ viewpoint and 18.20 ± 5.26 from the nurses’ point of view, with a significant difference (*P* < 0.0001). The lowest mean score of palliative needs in these two groups was observed in the economic dimension, with the significantly different (*P* = 0.006) scores of 14.12 ± 2.97 and 15.02 ± 2.37 from the patients’ and the nurses’ points of view, respectively. The difference between the mean scores of total palliative needs from the patient’s point of view (149.27 ± 15.01) and the nurses’ point of view (140.07 ± 13.12) was statistically significant (*P* < 0.0001) (Table 3).

**Table 3:** Comparison of the palliative needs of MS patients in patients ‘viewpoints and nurses’ viewpoints

Dimensions	From the nurse’s viewpoint Mean ± SD	From the patient’s viewpoint Mean ± SD	*P*-value
Physical	45.01 ± 8.22	42.58 ± 9.58	0.02^*^
Social	35.26 ± 5.10	39 ± 6.35	< 0.0001^*^
Spiritual	26.57 ± 3.72	31.24 ± 4.07	< 0.0001^*^
psychological	18.20 ± 5.26	22.31 ± 6.81	< 0.0001^*^
Economical	15.02 ± 2.37	14.12 ± 2.97	0.006^*^
Total Palliative needs	140.07 ± 13.12	149.27 ± 15.01	< 0.0001^*^

*Significant *p*-value *p* < 0.05

## Discussion

The study results showed that in this study, the highest average palliative score from the patients and nurses’ viewpoints was related to the physical dimension, followed by the social, spiritual, and psychological dimensions; the lowest mean score belonged to the economic dimension. Nevertheless, the mean scores in all domains were significantly higher from the patients ‘point of view than from the nurses’ point of view. It seems that patients have a greater understanding of their needs due to the suffering they have endured over the years, and therefore they have given a higher score to each of their needs. In line with our study, Forbes et al. group the needs of multiple sclerosis patients into seven categories: medical treatment, socio-environmental support and adaptation, enhanced care provision, information provision, rehabilitation treatments, non-professional care, and psychological support [29]. Conversely, McIlfatrick et al. assessing palliative care needs from the perspective of patients, self-employed people, and healthcare professionals, found that the main areas of need identified by their participants were social and psychological support, financial concerns, and the need for choice and information, and all participants believed that there was inequity between palliative care service provision for patients with cancer and non-cancer diseases [30]. Also, Lorefice et al. assessed the perceptions of patients and caregivers in a study and showed that the majority of the participants were satisfied with the medical staff. However, they expected to receive more psychological support and more information on MS [31]. Ponzio et al. conducted a study to accurately describe the essential support services they called ‘unmet care needs in patients with MS. All participants chose at least one health or social support requirement, and one of the most common health care needs chosen by them was psychological support [32]. Although in these studies, the greatest need was related to psychological needs, in our study, the greatest need of patients was relief of physical symptoms. The difference between our results and the results of other studies could be related to the difference in treatment facilities to relieve patient symptoms. Even the difference in the priority of palliative care need dimensions with the present study may have been influenced by the environment and different living conditions. Many Iranian people still live traditionally, resulting in fewer psychological problems for patients. These findings highlight the importance of achieving a balance between palliative care needs and patient-centered care. On the other hand, the importance of medical treatment to patients is in line with Maslow’s hierarchy of human needs, which asserts that physiological needs are fundamental needs and must be met before others. Therefore, symptom management takes primacy over all other needs [29].

The next palliative care needs in order of priority from the perspective of patients and nurses were social, spiritual, and psychological needs. In a similar study identifying palliative care needs of patients and their families in the neuro-ICU, Creutzfeldt et al. studied the potential ways to respond to those needs, and palliative care needs were identified in the vast majority of patients, and most of the needs were related to social support and basic needs based on care goals [33]. Potemkowski et al. aimed to identify the most important psychological and social needs of patients with MS in Poland to study 573 patients with MS and 220 of their spouses; this study used a similar questionnaire and, therefore, yielded similar results concerning the needs of MS patients [34]. The contradiction with the results of the present study seems to be because the study conducted in Poland did not measure all aspects of patients’ needs and only addressed the psychological and social needs of these patients. It seems that disability and cognitive impairment are predictors of withdrawal from social and leisure activities among MS patients and are strong stress indicators among relatives. Several studies have shown that social support is an important positive factor in the health-related quality of life in patients with MS [35–37].

In this study, the least essential palliative need was the economic dimension. Consistent with these results, Bahador et al. showed that the lowest average score for palliative care need was in the economic dimension [38]. Penrod et al. conclude that the patients receiving palliative care were less likely to be hospitalized in intensive care units, which led to a reduction in healthcare costs [39]. On the other hand, McIlfatrick et al. addressed one of the main areas of need identified by all participants, including patients, non-professional caregivers, and healthcare professionals was financial concerns and the need for choice and information [30]. The researchers stated that a decrease in income after receiving an MS diagnosis was reported by 56.2% of patients, with 37.1% experiencing a decrease greater than 20% in their income. 67% of patients used at least one financial coping strategy in response to the financial burden of treatment expenses. Furthermore, 50.4% of the participants decreased spending on leisure activities, and 34.8% decreased basic spending [40, 41]. MS patients, especially, faced considerable financial hardship due to the expensive treatments, higher rates of disability, and reduction in income. Higher costs for advanced imaging tests and increased cost-sharing have caused the financial burdens on MS patients to worsen [42].

These findings demonstrate that MS patients need to receive palliative care programs emphasizing the identification of palliative needs to ensure more satisfactory lives. Edmonds et al. showed that palliative care services had a positive effect on some of the key symptoms, and it is possible to provide services that address specific needs of MS patients [43]. Also, Solari et al. demonstrated that the symptom burden (POS-S-MS) significantly decreased in their home-based palliative approach group compared to the group receiving usual care [44]. Oishi et al. showed that uncertainty about the disease and lack of cooperation among health care professionals are barriers to effective care [21]. Therefore, to meet the needs of patients with severe MS, health services should be considered taking into account existing palliative care services and should be given greater importance among the health care providers [45].

The present study was limited by some factors. For instance, the data on the patients and nurses were of the self-report type. Another limitation of this study was that due to the high level of information, this article is not about the content but only about the amount of problems and needs.

## Conclusion

From the perspective of the patients with MS and their nurses, all patients with MS have palliative care needs in physical, social, spiritual, psychological, and economic dimensions, and patients should be able to access palliative care appropriate to their individual needs. Attention to these issues has important implications for the future planning and delivery of palliative care services.

It is essential to note the difference in the nurses’ and patients’ perceptions of palliative care needs. Health care providers must know the primary concerns and needs of patients as this can lead to the provision of quality care tailored to the needs of patients and may increase the feeling of well-being and improve the quality of life of patients. As a result, palliative care is an essential aspect of clinical nursing, affecting the diagnosis, interventions, assessments, and patient care schedules. Considering the patient’s needs and giving appropriate information and choices based on the patient’s desire are among the most critical factors.

## Data Availability

The datasets used and/or analyzed during the current study available from the corresponding author on reasonable request.
